# Radiofrequency-Assisted Liver Resection Versus Clamp-Crush Liver Resection: Protocol for an Updated Meta-Analysis and Systematic Review

**DOI:** 10.2196/13437

**Published:** 2019-08-20

**Authors:** Osman El-Koubani, Mark McCann, Daniel Christmas, Aakash Khanna, Alexander von Maydell

**Affiliations:** 1 Faculty of Medicine Imperial College London London United Kingdom; 2 Department of General Surgery Royal Infirmary Edinburgh Edinburgh United Kingdom; 3 Faculty of Medicine University of Aberdeen Aberdeen United Kingdom

**Keywords:** radiofrequency, hepatectomy, systematic review, meta-analysis

## Abstract

**Background:**

Malignancy of the liver has historically meant a poor prognosis and remains the second most common cause of cancer-related deaths globally. Traditionally, hepatectomy has utilized the clamp-crush technique; however, this is associated with high incidence of postoperative complications. Many novel techniques have been developed—radiofrequency ablation and transarterial chemoembolization; however, these are not applicable to numerous cases. Clamp-crush liver resection (CCLR) remains the gold standard. Radiofrequency-assisted liver resection (RFLR) is a technique that aims to reduce mortality through bloodless liver resection. A systematic review was previously performed on RFLR but the results neither recommended nor refuted the use of RFLR owing to the lack of sufficient evidence from well-designed randomized controlled trials (RCTs) at the time.

**Objective:**

The aim of the study is the meta-analysis and systematic review of recent studies comparing RFLR against CCLR.

**Methods:**

Articles comparing RFLR and CCLR that were published from 2014 until 2019 will be reviewed and relevant data will be extracted and statistically analyzed through Review Manager 5 (by the Cochrane Collaboration) together with the results of the previous meta-analysis.

**Results:**

Data collection is currently underway, with papers being screened. We hope to publish the results by the end of 2019.

**Conclusions:**

Given the high mortality rates currently associated with liver resection, it is imperative that novel surgical techniques are undertaken and investigated so we can improve best practice guidance and outcomes.

**International Registered Report Identifier (IRRID):**

DERR1-10.2196/13437

## Introduction

### Background

Malignancy of the liver has historically meant a poor prognosis for patients and remains the second most common cause of cancer-related deaths globally [1]. Many novel nonsurgical techniques for treating carcinoma of the liver have been developed, such as radiofrequency ablation or transarterial chemoembolization [2]. These techniques have shown promising results in their early usage. However, liver resection continues to be the primary intervention for management of benign and malignant liver lesions. Although advancements in techniques for resection have led to better morbidity and long-and-short-term mortality rates in recent years, only a few patients remain eligible for surgery, given the risks associated with surgery. Concerns regarding surgery are primarily related to intraoperative bleeding time and subsequent requirement of blood transfusion, both of which have been shown to lead to poor outcomes [3,4]. The risks associated with bleeding are especially pertinent as many patients with liver tumors have concurrent poor liver function.

Incidence of hepatocellular carcinoma (HCC) is predicted to rise to 22 million over the next few decades owing to rises in cirrhotic liver disease related to alcohol and viral hepatitis and Noncirrhotic Liver Disease related to Fatty Liver Disease [5]. Given these projections, further innovation in management of liver tumors is necessary to continue improving outcomes and access to treatment for patients. Standard methods for liver resection traditionally utilize the clamp-crush technique, which allows for parenchymal transection of the liver with reduced bleeding intraoperatively. The Pringle maneuver is commonly employed during liver surgery, which clamps the hepatoduodenal ligament, thus interrupting blood flow through the hepatic artery and portal vein. It does however often result in the risks previously specified such as bleeding, requirement for transfusion, and leaking of bile postoperatively, all of which affect mortality and morbidity. Patients with poor hepatic reserve are also at risk of hepatic reperfusion injury owing to occlusion of blood flow.

Recent advances in surgical technology have led to the development of devices utilizing radiofrequency. These devices do not require hilar dissection or blood flow occlusion, mitigating risks associated with blood loss and reperfusion injury. The devices coagulate viable tissue around the resection margin, allowing for subsequent excision with minimal blood loss. A meta-analysis in 2014 by Xiao et al [6] looked at 9 studies showing statistically significant reduction in intraoperative blood loss in patients when comparing radiofrequency-assisted liver resection (RFLR) with clamp-crush liver resection (CCLR). There was, however, no significant difference observed in requirements for blood transfusion and showed increased rates of bile leak and intra-abdominal abscess [6]. Therefore, Xiao et al [6] concluded that the evidence neither supported nor refuted the use of RFLR. Since this study, multiple additional studies comparing RFLR and CCLR have been published. One recent meta-analysis conducted by Reccia et al in 2017 [7] was specifically interested in laparoscopic radiofrequency liver resections. They concluded that this type of resection was a safe and feasible procedure for both benign and malignant liver disease associated with a reduction in blood loss and hospital mortality rate [7]. Laparoscopic RFLR, however, constitutes a small minority of laparoscopic liver resections [7], which itself is not standard treatment for patients with localized colorectal metastases or HCC [8]. This meta-analysis is therefore interested in assessing the efficacy of open RFLR against CCLR, which would be more representative of common practice. There is still no definitive work arguing in favor or against the use of RFLR; therefore, we are unable to design guidance on best practices with regard to liver resection. Given the potential benefit of bloodless liver resection, this meta-analysis will be undertaken to give an update on developments by including the most recent studies.

### Approach

A systematic review will be undertaken assessing the recent studies from 2014 until 2019 that compare RFLR with CCLR for all various types of devices that utilize radiofrequency energy for liver resection. The search strategy will therefore include any individual device that has evidence published comparing its efficacy to CCLR, such as Habib 4X System, Cool-tip System, Tissue Link, and Radionics. Relevant data from these studies and the meta-analysis by Xiao et al [6] will be extracted and a combined meta-analysis will be performed.

### Objectives

#### Population, Intervention, Comparison, and Outcome Framework

- Population: patients requiring liver resection for benign or malignant liver disease in both normal and cirrhosed liver.
- Intervention: RFLR.
- Comparison: CCLR.

#### Outcome Measure

- Primary outcome: intraoperative blood loss.
- Secondary outcomes: (1) operation time; (2) number of patients developing postoperative bile leak; (3) number of patients requiring blood transfusion; (4) number of patients developing intra-abdominal abscess; and (5) mortality at 30 days.
- Study design: all human study types (randomized and nonrandomized) comparing RFLR and CCLRs.

## Methods

The source databases were as follows: (1) PubMed (2) Ovid (3) EMBASE and (4) Cochrane.

### Search Strategy

Mirroring the search strategy employed by Xiao et al [6], we will search in the fields of *Abstract* and *Title* radiofrequency together with *hepatectomy, liver resection, liver transection*, or *liver surgery* as keywords, in addition to using the medical subject headings term *hepatectomy* with subheading *mortality*. The search will begin from December 2012, the date which represents when the previous systematic review by Xiao et al was performed [6]. No upper date limit will be used. There will be no language restrictions for the search.

#### Search String

(((radiofrequency[All Fields] AND “hepatectomy/mortality”[Mesh Terms]) OR (radiofrequency[All Fields] AND liver resection[Title/Abstract])) OR (radiofrequency[All Fields] AND liver transection[Title/Abstract])) OR (radiofrequency[All Fields] AND liver surgery[Title/Abstract]) AND (“2012/12/01”[PDAT]: “3000/12/31”[PDAT]) AND “humans”[MeSH Terms]

### Eligibility

#### Inclusion Criteria

The inclusion criteria were as follows: all studies published as full-text articles from peer-review journals comparing RFLR and CCLR in which at least data from one of the quantitative outcomes mentioned are reported. These studies had to have more than 20 patients included in them.

#### Exclusion Criteria

1. Nonhuman studies.
2. No control group.
3. Publication not available in English.
4. Review articles.
5. Letters and editorial comments.
6. Case reports.

### Screening

We will use the Systematic Review Facility Web-based screening tool to screen the title and abstract of each paper that is identified in our literature search. This will be done by 2 independent reviewers (EO and MM) assessing each paper’s eligibility against our inclusion and exclusion criteria. A third reviewer (CD) will then confirm the appropriateness of extracted papers.

### Quality Assessments

Quality assessment will mirror similar methodologies to Xiao et al [6] to accurately compare the quality of all studies. Randomized controlled trials (RCTs) and nonrandomized studies will be assessed as shown below.

#### Randomized Control Trials

The quality of all RCTs will be assessed using a modified Jadad score, comprising the following 5 variables [9]:

1. Randomization.
2. Generation of random numbers.
3. Details of the double-blinding procedure.
4. Information on withdrawals.
5. Concealment of allocation.

One point is allocation for each variable from the criteria that a study includes, totaling a maximum score of 5.

#### Nonrandomized Controlled Trials

The quality of all non-RCTs will be assessed using the modified Newcastle-Ottawa scale that is recommended by the Cochrane Collaboration [10], comprising the following 3 variables [11]:

1. Patient selection—comprising 4 items, worth one point each.
2. Comparability of study groups—comprising 1 item, worth up to 2 points.
3. Assessment of outcomes—comprising 3 items, worth one point each.

Therefore, the maximum score is 9, and the score is represented by stars. Studies labeled with 6 or more stars will be considered to be of high quality.

### Statistical Analysis

Analysis of the data will be done through Review Manager 5, provided by the Cochrane Collaboration. All included studies for each outcome measure will be presented graphically and plotted on a forest plot. The comparison of dichotomous outcomes will be through their odds ratios with 95% Cis. Continuous variables will be compared through their weighted mean differences with 95% CI. Any *P* value <.05 will be deemed statistically significant. Heterogeneity will be assessed using I^2^ values. Low heterogeneity will be defined as an I^2^ value of 50% or less, in which case the fixed-effects model will be used. If there is high heterogeneity between studies, the random-effects model will be implemented. To identify patient groups that may benefit from this treatment, for example, patients with cirrhotic livers, a subgroup analysis will also be undertaken for more homogeneous studies.

## Results

Data collection is currently underway, with the screening process being undertaken. We will then begin data analysis, with the expectation to publish results and a full manuscript by the end of 2019.

## Discussion

Given the growing complexity of patients, a procedure that reduces the known risks associated with high morbidity and mortality is desirable. Although intraoperative blood loss and subsequent blood transfusions are traditionally expected within surgery, in relation to liver resection, it is associated with poor outcomes. The crucial period in minimizing blood loss is during division of the liver parenchyma. Surgical techniques such as radiofrequency can theoretically eradicate bleeding during this period, leading to bloodless liver resections, thus improving outcomes for patients. However, if new surgical techniques such as RFLR cause a rise in postoperative complication rates, for example, bile leak and infection, they will not be favorable alternatives to traditional CCLR. Given the poor outcomes currently associated with liver resection, it is imperative that new best practice surgical techniques are undertaken.

## Abbreviations

CCLR: clamp-crush liver resection
HCC: hepatocellular carcinoma
RCT: randomized controlled trials
RFLR: radiofrequency-assisted liver resection
